# The chemical cue tetrabromopyrrole induces rapid cellular stress and mortality in phytoplankton

**DOI:** 10.1038/s41598-018-33945-3

**Published:** 2018-10-19

**Authors:** Kristen E. Whalen, Christopher Kirby, Russell M. Nicholson, Mia O’Reilly, Bradley S. Moore, Elizabeth L. Harvey

**Affiliations:** 1 Department of Biology, Haverford College, Haverford, PA USA; 2 Skidaway Institute of Oceanography, University of Georgia, Savannah, GA USA; 3 Scripps Institution of Oceanography, University of California at San Diego, La Jolla, CA USA

## Abstract

Eukaryotic phytoplankton contribute to the flow of elements through marine food webs, biogeochemical cycles, and Earth’s climate. Therefore, how phytoplankton die is a critical determinate of the flow and fate of nutrients. While heterotroph grazing and viral infection contribute to phytoplankton mortality, recent evidence suggests that bacteria-derived cues also control phytoplankton lysis. Here, we report exposure to nanomolar concentrations of 2,3,4,5-tetrabromopyrrole (TBP), a brominated chemical cue synthesized by marine γ-proteobacteria, resulted in mortality of seven phylogenetically-diverse phytoplankton species. A comparison of nine compounds of marine-origin containing a range of cyclic moieties and halogenation indicated that both a single pyrrole ring and increased bromination were most lethal to the coccolithophore, *Emiliania huxleyi*. TBP also rapidly induced the production of reactive oxygen species and the release of intracellular calcium stores, both of which can trigger the activation of cellular death pathways. Mining of the Ocean Gene Atlas indicated that TBP biosynthetic machinery is globally distributed throughout the water column in coastal areas. These findings suggest that bacterial cues play multiple functions in regulating phytoplankton communities by inducing biochemical changes associated with cellular death. Chemically-induced lysis by bacterial infochemicals is yet another variable that must be considered when modeling oceanic nutrient dynamics.

## Introduction

Naturally produced polyhalogenated organic compounds are pervasive in the marine environment and have been isolated with regularity from marine biota^1^, including sponges and associated cyanobacteria^2^, macroalgae, invertebrates^3^, and bacteria^4,5^. In particular, the ocean provides a large reservoir of bromide, making bromination a diagnostic feature of marine natural products in contrast to terrestrial systems^6^. Polybrominated aromatic compounds, including a diverse suite of polybrominated pyrroles, have been an area of particular interest for their ecological impacts^7^, influence on coral larval settlement and metamorphosis^8,9^, and selectivity for physiological targets^10,11^. The recent discovery of the conserved biosynthetic gene cluster for synthesis of polybrominated aromatic compounds in marine γ-proteobacteria suggests that these microorganisms may be key producers of these polybrominated compounds in the ocean^6^.

The marine bacteria harboring this biosynthetic pathway belong to a genus of γ-proteobacteria known for their ubiquity in the water column^12^, on marine surfaces, and in association with eukaryotic hosts^8,9,13,14^. Members of the γ-proteobacteria include the genus *Pseudoalteromonas*, which constitutes 0.5–6.0% of the bacterial species found globally and are frequently isolated from biofilms covering the surface of marine organisms^12^. Representative species within this genus are motile, rod-shaped, and known to be pathogenic or opportunistic^15^. Beyond their importance in biofilm formation, *Pseudoalteromonas* spp. can display anti-bacterial and algicidal activity, and impact settlement, germination, and metamorphosis of invertebrate species^15–17^.

Members of the genus *Pseudoalteromonas* have been of great interest pharmacologically due to its metabolite-producing capacity, however, its interactions with other domains of life have continued to establish the ecological significance of this genus in marine systems^15^. *Pseudoalteromonas* spp. can display predatory-like behavior^15^ and are known produce a diverse suite of compounds including antimicrobial alkaloids, polyketides, peptides and extracellular serine proteases, metalloproteases and less characterized proteolytic substances thought be to capable of suppressing competitive microorganism interactions^18^. In general, *Pseudoalteromonas* spp. are frequently implicated as producers of extracellular products that cause phytoplankton mortality^15,19,20^. These bacteria have been found to produce a suite of biologically active metabolites that include a range of polybrominated pyrroles^17^. One brominated compound produced by *Pseudoalteromonas* spp., tetrabromopyrrole (TBP), has been shown to exhibit antibiotic activity against human and marine bacteria, and may alter bacterial community composition within marine biofilms^21^. Additionally, TBP has been shown to be the causative agent responsible for inducing larval settlement and metamorphosis without attachment in coral larvae^8,9^, as well as contribute to the immobilization or death of pre-competent (1–4 day old) coral larvae^22^. However, despite its known importance to the ecology of scleractinian corals, the impacts of TBP on other marine organisms, such as phytoplankton, have yet to be elucidated.

Interactions between phytoplankton and bacteria play a central role in mediating biogeochemical cycles and microbial trophic structure in the ocean. These interactions can be complex – both beneficial^23^ and/or detrimental to phytoplankton growth dynamics^24^. Given that phytoplankton grow exponentially when nutrients are non-limiting; even slight decreases in growth rates will have the potential to significantly influence their population dynamics, ultimately influencing large-scale oceanic processes. The impact of γ-proteobacteria, including *Pseudoalteromonas* spp., on both phytoplankton growth^25^ and phytoplankton mortality^26–29^ has been documented in the literature. However, only recently with the advancement of technology have we been able to identify many of the causative agents responsible for inducing mortality^30–33^. As compounds are described, additional physiological and biochemical assays are needed to diagnose the underlying molecular targets and mechanisms responsible for algal cell death.

Our previous work investigating algicidal bacteria indicated that *Pseudoalteromonas piscicida* (A757) produced two compounds with distinct algicidal properties^30^. The first compound to be described, 2-heptyl-4-quinolone (HHQ), a diffusible signaling molecule used by bacteria to communicate in a density-dependent manner, caused static growth in the globally distributed coccolithophore, *Emiliania huxleyi*, rather than immediate cell lysis upon exposure. In contrast, here we describe the identification of a second compound, TBP, whose activity resulted in immediate cell lysis within 24 h of exposure. We investigated the effect of TBP on a seven different phytoplankton species including diatoms, cryptophytes, and coccolithophores; and for all species, TBP exposure induced algal mortality within 24 to 48 hrs. We also employed various diagnostic fluorescent cell assays to probe for stress-related cellular effects in *E*. *huxleyi* in response to TBP exposure. In addition, we examined the pervasiveness of genes involved in the halopyrrole biosynthesis in the water column by mining metagenomic databases in order to explore the biogeography of heterotrophic bacteria with this biosynthetic capacity, and facilitate comparison with the distributions of TBP-susceptible phytoplankton species. Ultimately, this research will provide new insights into how bacterially-derived compounds can influence and potentially mediate phytoplankton population dynamics and oceanic biogeochemical cycles.

## Results

### Tetrabromopyrrole Identification

Our original investigation of the algicidal activity of *P*. *piscicida* exometabolome indicated that this species of bacteria produced at least two separate compounds capable of inducing mortality in *Emiliania huxleyi*. A previous publication^30^, detailed the response of live cells and cell filtrate of *P*. *piscicida*, as well as the description of the discovery of one of the newly discovered algicidal compound, 2-heptyl-4-quinolone (HHQ). Here, bioassay-guided fractionation was used to isolate a second bacterial compound and the structure of the active fraction was investigated with high-resolution mass spectra experiments. Analysis of the purified active compound in negative ionization mode identified peaks at *m/z* 377.67, 379.67, 381.67, 383.67, 385.66 (1:4:6:4:1) indicating a tetrabrominated compound. HRMS (ESI): *m/z* calculated for C_4_NBr_4_ ([M-H]^1−^), 377.6770; found 377.6768 (Supplementary Fig. S1). This compound was confirmed to be tetrabromopyrrole by comparison with literature values and retention times to that of an authentic standard. Moreover, the presence of TBP in A757 had previously been identified in a high-resolution profiling of *P*. *piscicidia* (A757) exometabolome^4^.

### Dose-Response Experiments with Phytoplankton

**Figure 1:**
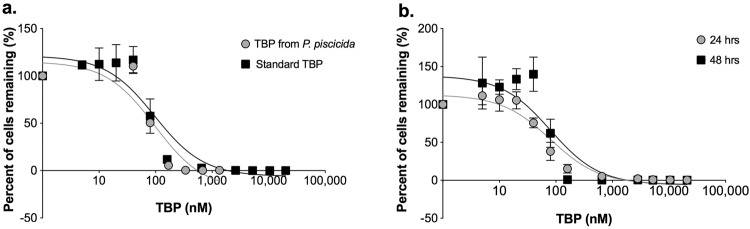
Response of *Emiliania huxleyi* to TBP. (**a**) A comparison of the dose response of *E*. *huxleyi* to TBP isolated from *P*. *piscicida* (gray) and TBP standard (black), see Table 1 for full statistical comparison of the curves. (**b**) A comparison of the percent of *E*. *huxleyi* cells remaining after 24 (gray) or 48 h (black) exposure to TBP. The lack of a significant difference between these two curves suggests that the majority of *E*. *huxleyi* mortality in response to TBP occurred during the first 24 h of exposure. Each symbol represents the mean of three independent replications ± the standard deviation.

**Figure 2:**
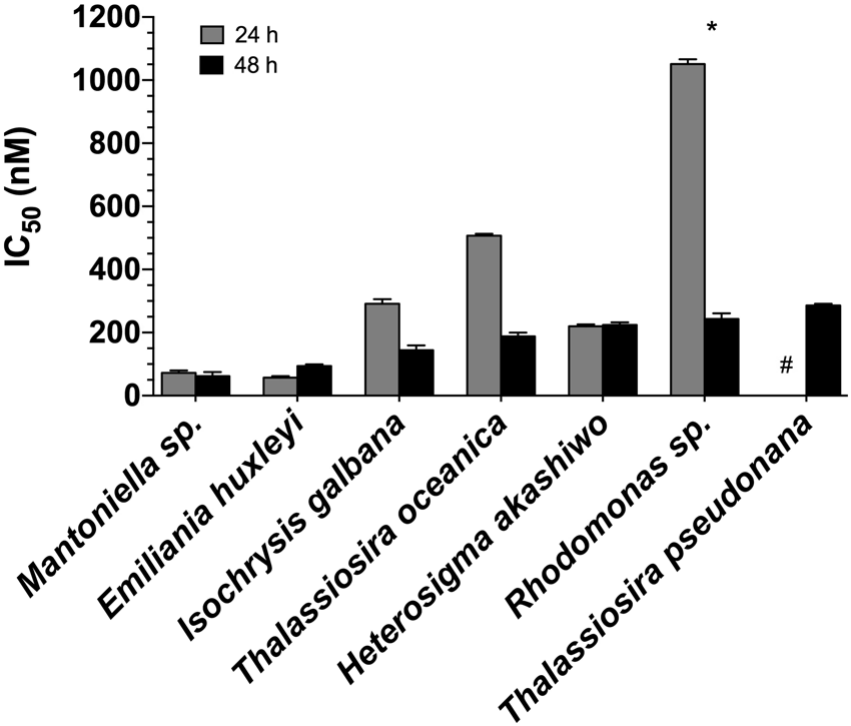
Response of various phytoplankton species to TBP. A comparison of TBP inhibitory concentrations (IC_50_) for seven phytoplankton species derived from dose-response curves, calculated after 24 (gray) and 48 h (black) of exposure to TBP. Each symbol represents the mean of three independent replications ± the standard deviation. No IC_50_ could be calculated for *T*. *pseudonana* after 24 h of exposure (hashtag), as no mortality was observed below 5225 nM. *Rhodomonas* sp. was the only species tested to have a significantly lower IC_50_ value after 48 h of exposure, relative to the IC_50_ value observed after 24 h. See Table S1 for additional IC_50_ results.

Dose-response experiments with *E*. *huxleyi* determined the inhibitory concentration (IC_50_) for the isolated pure compound to be 99 ± 10 nM, which was not significantly (p = 0.89) different from the IC_50_ obtained when the algae was exposed to the TBP standard (94 ± 5 nM; Fig. 1a, Table 1). Furthermore, there was no significant difference (p = 0.19) in the IC_50_ calculated for *E*. *huxleyi* when exposed to TBP for 24 or 48 h, indicating that the onset of the observed mortality occurred rapidly within the first 24 h (Fig. 1b). For all phytoplankton species examined, IC_50_ values ranged from 57 ± 5 to 1051 ± 15 nM after 24 h of exposure to TBP (Fig. 2, Table S1). IC_50_ values calculated for TBP exposure at 24 h and 48 h were not statistically different from one another with the exception of two species, *Rhodomonas* sp. and *T*. *pseudonana* (Table S1). This suggests that for most species examined, mortality occurred within the first 24 h of the experiment. For *Rhodomonas* sp., IC_50_ values were significantly higher (p = 0.01) following 24 h of exposure to TBP compared to 48 h. For *T*. *pseudonana*, no IC_50_ could be calculated after 24 h, as mortality was only observed at the highest concentration tested (20,900 nM), therefore we could not make a comparison between the 24 and 48 h time points. However, mortality was observed for *T*. *pseudonana* at 286 ± 5 nM after 48 h of exposure to TBP (Table S1), indicating that this phytoplankton was susceptible to the effects of TBP.

**Table 1:** IC_50_ values for TBP (isolated and standard).

Compound	IC_50_ (ng mL^−1^)	IC_50_ (nM)	R^2^	95% CI (nM)	p-value
A757 isolated	38 (4)	99 (10)	0.84	54–191	0.89
TBP standard	36 (2)	94 (5)	0.89	60–151	

Statistical comparison of the dose-response curves and inhibitory concetrations (IC_50_) values generated for *Emiliania huxleyi* exposed to the pure TBP extracted from a *Pseudoalteromonas piscicida* culture and an authentic standard. Values in parentheses are one standard deviation of the mean.

*E*. *huxleyi* cells that had been exposed to TBP concentrations at 165 nM or below were able to recover once removed from TBP exposure (Supplementary Fig. S2). After 72 h in TBP-free, f/2 –si media the growth rate of *E*. *huxleyi* exposed to 165 nM of TBP was 0.40 ± 0.06 d^−1^, which did not significantly differ from rates calculated for DMSO controls (0.45 ± 0.07 d^−1^) or cell only (0.43 ± 0.04 d^−1^) controls (p = 0.94) lacking either TBP or DMSO. In contrast, *E*. *huxelyi* that had been exposed to TBP concentrations of 650 nM or higher exhibited significantly lower growth rates (p = 0.03; 0.03 ± 0.09 d^−1^ for 650 nM) or no measurable growth (concentrations >650 nM) when tested in recovery assays.

**Figure 3:**
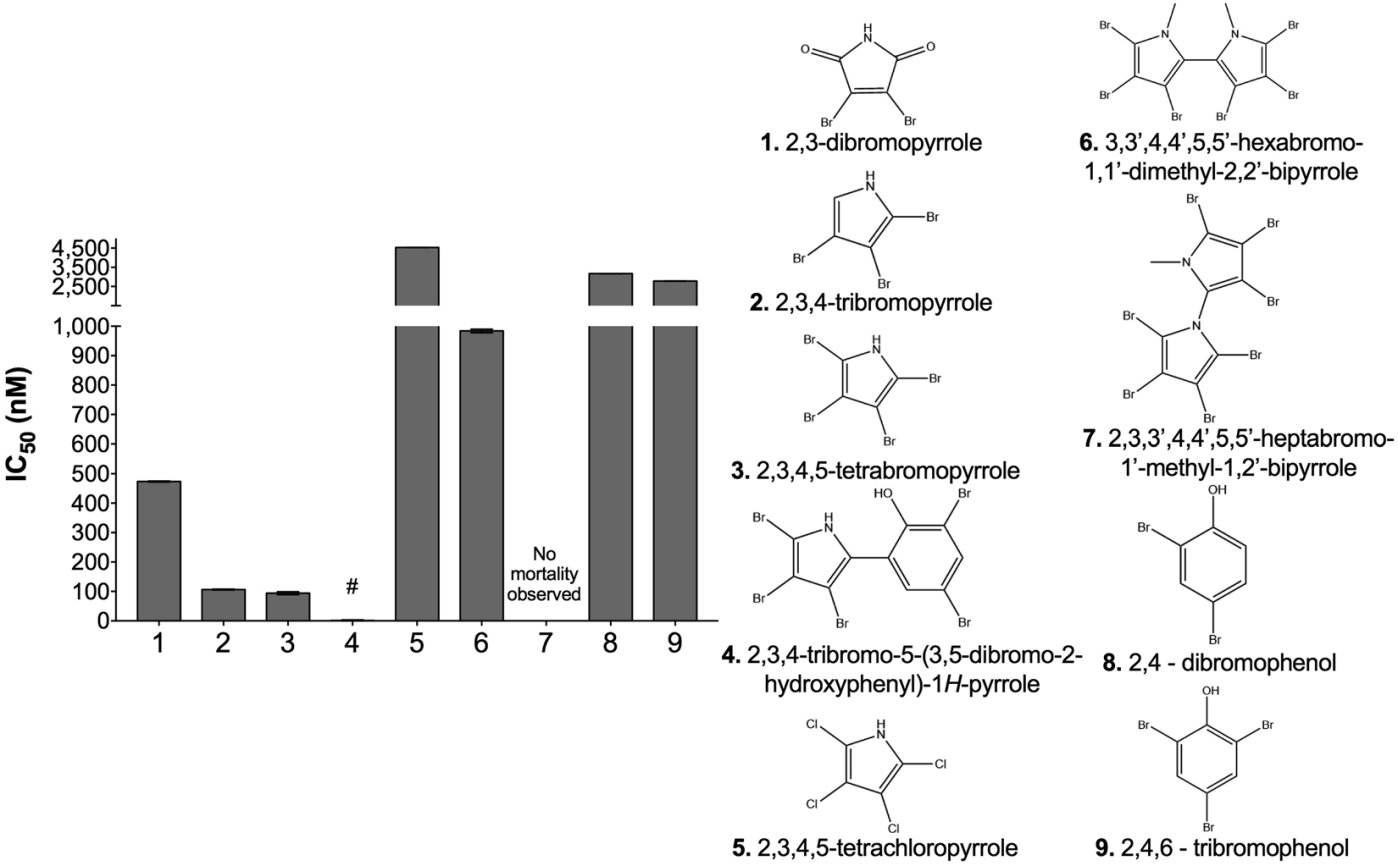
Response of *E*. *huxleyi* to a diverse range of organohalogens. The inhibitory concentrations (IC_50_) for *E*. *huxleyi* when exposed to seven different halogenated compounds for 72 h. The IC_50_ for 2,3,4-tribromo-5-(3,5-dibromo-2-hydroxyphenyl)-1*H*-pyrrole (hashtag) was 1.35 ± 1.71 nM and not visible in relation to the IC50′s of the other compounds shown in the bar graph. No reduction in growth was observed for *E*. *huxleyi* cells when exposed to 2,3,3′,4,4′,5,5′-heptabromo-1′-methyl-1,2′-bipyrrole. Each bar represents the mean of three independent replications ± the standard deviation.

In order to determine how the degree and type of halogenation, as well as the aromatic ring structure affects algicidal activity, we exposed *E*. *huxleyi* to eight additional marine-derived organohalogens (Fig. 3). In contrast to tetrabromopyrrole, the IC_50_ value for tetrachloropyrrole was an order of magnitude higher (4530 ± 3 nM), suggesting the presence of chlorine atoms on a pyrrole backbone was not sufficient to recapitulate earlier algicidal activity. Moreover, no relationship between the degree of bromination and *E*. *huxleyi* susceptibility was observed. However, when ring structure and degree of bromination were both considered, those compounds containing a single pyrrole ring and at least one bromine were found to display significantly higher (p < 0.001 for all comparisons) algicidal activity (i.e., had lower IC_50_ values) against *E*. *huxleyi*, with IC_50_ values ranging from 1.3 ± 1.7 nM to 473 ± 1.4 nM. Additionally, as the degree of bromination increased among single pyrrole containing compounds, the IC_50_ values decreased with pentabromopseudilin demonstrating the lowest IC_50_ recorded at 1.3 nM, which was significantly lower (p < 0.001 for all comparisons) then what was observed for tetra-, tri- and di-bromopyrrole at 94, 106, 473 nM, respectively. Together, these results indicate that, of the compounds tested, a combination of both a single pyrrole ring and increased bromination are most lethal to *E*. *huxleyi*.

### Impact of TBP on calcium and reactive oxygen species signaling

**Figure 4:**
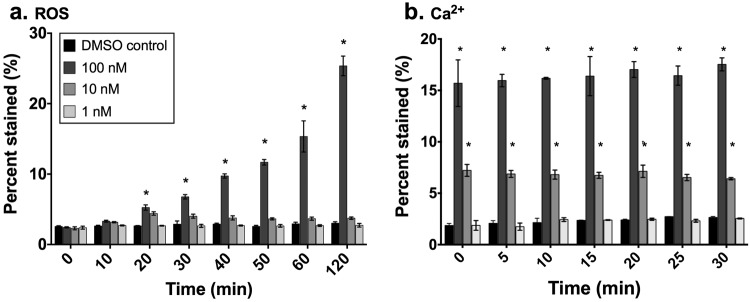
Endogenous ROS production and intracellular calcium release in response to TBP. ROS (**a**) or Ca^2+^ (**b**) production in *E*. *huxleyi* after the addition of 1 nM (light gray), 10 nM (medium gray), or 100 nM (dark gray) TBP. Black bars represent DMSO solvent controls. Treatments denoted with an asterix were significantly different from the DMSO control for the given time point. Each symbol represents the mean of three independent replications ± the standard deviation.

Flow cytometry in combination with diagnostic staining was used to observe physiological stress responses of *E*. *huxleyi* when exposed to TBP (Fig. 4). Intracellular reactive oxygen species (ROS) and intracellular Ca^2+^ concentrations increased significantly in a dose-dependent manner when *E*. *huxleyi* cells were exposed to both 10 nM and 100 nM of TBP. When *E*. *huxleyi* was exposed to 100 nM TBP, the percentage of cells that stained positive for intracellular reactive oxygen species production, as measured by the dye CM-H_2_DCFDA, was significant (p < 0.001) as soon as 20 min post exposure, relative to the DMSO control. This treatment displayed measured accumulation for at least 120 min post exposure, however, no significant ROS accumulation was observed for *E*. *huxleyi* exposed to 1 or 10 nM TBP. Intracellular Ca^2+^ accumulation, as measured by Fluo-4 AM dye, was instantaneous and significantly different (p < 0.001 for all time points) relative to the DMSO control for *E*. *huxleyi* cells exposed to 10 and 100 nM TBP. The proportion of the population that stained positive for intracellular Ca^2+^ was consistent over the entire duration of the 30 min observation period following TBP exposure. No significant response was observed in either fluorescent assay when *E*. *huxleyi* was exposed to 1 nM of TBP.

### Exploring the biogeography of genes involved in bacterial biosynthesis of halogenated pyrroles

**Figure 5:**
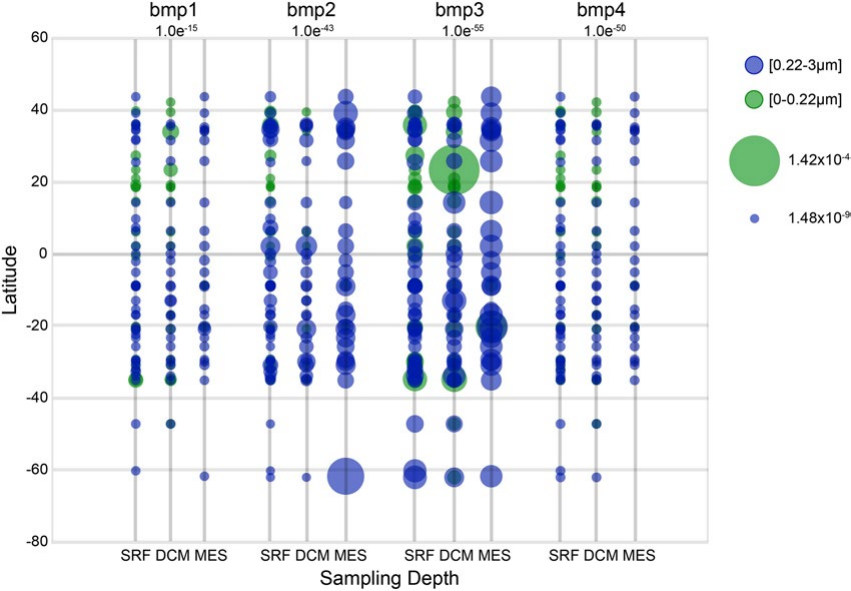
Biogeographical assessment of TBP biosynthesis genes. Genes with homology to pseudoalteromonad TBP biosynthesis genes are ubiquitously present in waters globally. The Ocean Gene Atlas^35^ was mined using protein sequences from *P*. *piscicida* (A757) *bmp1* to *bmp4* genes, shown to be involved previously in TBP synthesis^34^. The circle diameter indicates the percent of genes within an environmental sample matching the query which ranges from 0.000142% to 0.00000000148% within the given e-value cutoffs. Circle color indicates the size fractionation applied to the sample. Gene abundances with homology to each query are shown for subsurface (SRF), deep chlorophyll maximum (DCM), and mesopelagic (MES) depths.

Recently, the molecular basis for tetrabromopyrrole biosynthesis was described for *Pseudoalteromonas piscicida* (A757)^34^. Sequencing of the isolate revealed a conserved four-gene locus encoding the enzymes involved in TBP complete biosynthesis which is identical to the *bmp* gene cluster found in *Marinomonas mediterranea*. Moreover, a single flavin-dependent halogenase (Bmp2) is responsible for adding all four bromine atoms to TBP. Using the Ocean Gene Atlas (OGA) web service, we queried the protein sequences from *P*. *piscicida A757_bmp1–4* against the Ocean Microbial Reference Gene Catalog (OM-RGC) comprising 40 million non-redundant mostly prokaryotic gene sequences associated with both the Tara Oceans and Global Ocean Sampling (GOS) expeditions^35^. Using this environmental genomics datasets and companion biogeochemical profiles, we mined the OM-RGC database to determine the geolocalized abundances of all four TBP biosynthesis genes (*bmp1–4*) to gain additional insight into the pervasiveness of these genes within an environmental context. Using the Ocean Gene Atlas we were able to describe quantitatively the geographic distribution, co-variation with environmental features, and taxonomic distribution of environmental sequences sharing significant homology with *P*. *piscicida A757_bmp1–4*. The OGA platform identified 15, 53, 428, 119 homologs to *bmp1* to *bmp4*, respectively, using e-value threshold values of 1.0e^−15^, 1.0e^−43^, 1.0e^−55^, 1.0e^−50^, respectively. All four genes implicated in TBP biosynthesis showed similar abundances at all latitudes examined (Fig. 5), with *bmp3* gene homologs showing slightly higher abundances at all depths examined. When gene abundances were plotted by biogeophysical parameters (oxygen, nutrients, temperature, pH, distance), no obvious trends were observed except for distance from the shore (Supplementary Fig. S3). All homologs were found primarily within 10 km from shorelines. The abundance weighted taxonomic distribution of homolog sequences in all samples indicated that for *bmp1* gene homologs, the majority mapped to γ-proteobacteria (40%) including *Marinomonas* sp. with 8% mapping to cyanobacteria representatives including the symbiotic *Acaryochloris* sp. This finding is consistent with the presence of *bmp1* gene having been found in the marine cyanobacterial symbionts including *Prochloron didemni* P-2 Fiji^6^. Homologs to *bmp2* from *P*. *piscicida* (A757) originate mostly from the bacteria phyla Verrucomicrobia (64%) represented primarily by *Chthoniobacter* (33%) and *Verrucomicrobales* (18%), both of which have been identified as containing flavin-dependent halogenases in screening of metagenomic libraries derived from environmental samples^36^. Additionally, 3% of the gene abundances come from the phyla Proteobacteria with the majority described as unclassified and a quarter identified as *Marinomonas* sp. Gene abundances mapping to homologs of bmp3 were primarily in the Proteobacteria phyla (57%) with the majority within the γ-proteobacterial (35%) of which 6% were *Pseudomonas* sp. and 3% were *Pseudoalteromonas* sp., including *P*. *piscicida* contributing 0.09% of all bacteria. Finally, for *bmp4* homologs, 4% of all bacterial homologs mapped to *P*. *piscicida* specifically, with the majority of representatives (34%) mapping to γ-proteobacterial in general.

## Discussion

This study describes the isolation and identification of tetrabromopyrrole (TBP) from the antagonistic marine bacteria, *Pseudoalteromonas piscicida* (A757) and demonstrates the fast-acting algicidal activity of this compound on a diverse assemblage of phytoplankton species. When tested against seven diverse phytoplankton strains, TBP negatively impacted the growth of all species to varying degrees of severity within 24–48 h of exposure. The rapid mortality observed here suggests that phytoplankton do not need to be co-located with TBP producing bacteria for long periods of time in order to be impacted, and that TBP is not restrictive in its toxicity. While all phytoplankton species did experience mortality in response to TBP exposure, there was variability in the magnitude of IC_50_ values observed. Species-specific variability in the effects of toxins on plankton is common^37^, and the variability in the responses observed here may be related to compensatory mechanisms of TBP resistance. For example, organisms that share evolutionary/biogeographic history with a toxin producer can develop tolerances to the produced toxin^38^, and/or implore cellular mechanisms of detoxification^39^. However, the mechanisms that phytoplankton may use to protect against the effects of TBP are unknown at this time. Recovery experiments indicated that algal revival from TBP exposure was possible only when the concentration of TBP neared or was lower than the IC_50_. These results combine to highlight the irreversible toxicity of TBP to a wide range of phytoplankton species, directly mediating phytoplankton population dynamics.

The range of IC_50_ values calculated for TBP in the experiments presented here fall within a similar range of concentrations that have been previously shown to induce coral larval settlement^8^. Furthermore, the algicidal activity of TBP (IC_50_ = 36 ng mL^−1^ for a haptophyte) is an order of magnitude lower than the activity of the γ-proteobacterium-produced pyrrolylpyrromethane algicide, prodigiosin (LD_50_ = 2.24 µg mL^−1^ for the haptophyte, *Phaeocystis*)^28,40^. While natural concentrations of TBP in the phycosphere are unknown, the concentrations used in this study are similar to other bacterially-derived signaling molecules, including acyl-homoserine lactones, that have been measured at nanomolar concentrations in the aqueous phase of biofilms^41^. Moreover, given the propensity of *Pseudoalteromonas* spp. to form biofilms^42^ and the presence of TBP production in biofilm-forming *Pseudoalteromonas* spp.^8^, it is not unlikely for the concentrations of TBP to mirror other bacterially-derived metabolites in biofilms. With the ability of phytoplankton to host a diverse bacterial consortium on their surfaces^43^, there is the potential for phytoplankton to encounter TBP *in situ* at concentrations that induce mortality.

Beyond mortality, we found that exposure to TBP at 10 and 100 nM induced reactive oxygen species (ROS) and intracellular calcium (Ca^2+^) release. Release of ROS and Ca^2+^ in response to brominated compounds has been observed previously, for example, hydroxylated polybrominated diphenyl eithers (OH-PBDEs), are known to cause oxidative stress and induce developmental neurotoxicity within 24–28 hours of exposure in zebrafish^44^, and induce reactive oxygen species (ROS) production in avian cell lines^45^. Moreover, studies have indicated that PBDEs and their hydroxylated metabolites can promote Ca^2+^ release from intracellular stores via activation of ryanodine receptors (RyR) resulting in chronic Ca^2+^ leakage and depletion of endoplasmic reticulum stores thereby altering Ca^2+^ homeostasis^46^. Ryanodine receptors along with genetically related inositol-1,4,5-triphosphate receptors (ITPR) are found within the same protein superfamily and affect Ca^2+^ release from internal cellular stores^47^. A review of the evolution of calcium release channels in diverse eukaryotic phyla indicated that *E*. *huxleyi* genome has an inositol 1,4,5-triphosphate receptor (ITPR) containing a protein architecture characteristic of canonical metazoan members of this superfamily^47^. Moreover, a recent study found that halogenated pyrroles, including TBP of marine origin, were modulators of mammalian ryanodine receptors^48^. These authors also noted that the pyrrole moiety at 2-position on the diterpenoid ryanodine was essential for both high-affinity binding to RyR1 and conferring toxicity in mice. Interestingly, when we examined the pervasiveness of nine organohalogen’s ability to induce mortality in *E*. *huxleyi*, our results demonstrated that the most toxic compounds possessed a single pyrrole moiety with a high degree of bromination. When assessing an array of organohalogens, Zheng *et al*. 2018 found that polybromination of the pyrrole is essential for RyR1 activity, and further noted that brominated rather than chlorinated synthetic derivatives were more efficacious suggesting that steric bulk was important for conferring activity toward ryanodine receptors. Similarly, we found the IC_50_ for tetrachloropyrrole against *E*. *huxleyi* to be ~45-fold higher than for tetrabromopyrrole suggesting similar steric factors maybe in play as well.

In sum, the toxicity of polyhalogenated compounds appears to be mediated in part by oxidative stress resulting in the altered activities of key anti-oxidant defense proteins and calcium dynamics that regulate a diverse array of cellular functions. Further, both ROS and Ca^2+^ have been implicated as part of a stress-signaling cascade in marine phytoplankton, and are induced in response to a variety of stressors, at times leading to programmed cell death (PCD)^49^. The production of oxidative radical species like hydrogen peroxide and superoxide, and associated downstream responses are central to regulating algal cell fate by altering redox balance^50^. Several environmental stimuli have now been shown to increase ROS production in phytoplankton including thermal stress^51^, viral infection^52^, hydrogen peroxide exposure^53^, trace metal availability^54^, phytoplankton-derived infochemicals^55^, and bacterial algicide exposure^56^. Concomitant with ROS production, specific environmental stresses can also trigger intracellular Ca^2+^ release and nitric oxide (NO) generation leading to cellular death^50,57^. It has been suggested that non-stressed phytoplankton cells in proximity to damaged cells can sense diffusible sublethal conspecific-derived reactive infochemicals and induce, in a coordinated fashion, calcium- and NO-based signaling systems as a mechanism of self-protection^57^. In the case of TBP exposure, it would be fruitful to explore if chemically wounded phytoplankton cells releasing ROS and/or Ca^2+^ and nitric oxide (NO) could alert a similar surveillance system in non-stressed bystanders, or in turn, sublethal exposure to TBP could sensitize cells to induce protective mechanisms against programmed cell death. Future studies can be imagined that assess the physiological status of both chemically naïve bystanders and phytoplankton that have been exposed chronically to sublethal doses of TBP, and interrogate if and how the hallmarks of PCD are influenced.

Using the newly available Ocean Gene Atlas^35^ platform, we also present the first evidence for the global distribution of genes involved in TBP biosynthesis. By searching the database with protein queries from A757 we were able to confirm that each of the four conserved genes encoding enzymes involved in TBP biosynthesis were distributed throughout the water column (i.e., subsurface, deep chlorophyll maximum, and mesopelagic). Similar patterns in gene distribution have also been observed for other bacterial signaling pathways, with high levels of genetic diversity in quorum sensing autoinducers observed across the oceans^58^. Most of the observations of the TBP biosynthetic genes of interest examined here were restricted to coastal environments (<10 km from shore). However, without additional information it is impossible to distinguish if this higher abundance near the coast is driven by the decrease in bacterioplankton abundance that is often observed moving from shore to more open ocean^59,60^ or driven by interactions. Overall, this resource provided us with additional molecular clues as to the geographic underpinnings of the bacteria with organohalogen synthesis capabilities and how their distribution patterns might overlap with phytoplankton species. Further, this information can help to construct and constrain future investigations into the proclivity of TBP production in the ocean.

## Conclusions

The marine environment is complex and nutrients are typically ephemeral and contained in micro-patches^23^. Classically, marine bacteria have been categorized as those that either actively seek nutrient ‘hot spots’ and exploit them leading to enhanced growth^61^ or those that are more passive and have strategies to wait to encounter a nutrient windfall^62^. Herein, we demonstrate that bacteria can chemically manipulate the fate of phytoplankton. The rapid algal mortality observed in response to TBP exposure is fundamentally different from the static growth observed when phytoplankton are exposed to another compound produced by *P*. *piscicida*, 2-heptyl-4-quinolone (HHQ)^30^, which does not elicit ROS production or intracellular Ca^2+^ release in *E*. *huxleyi* (Whalen *et al*. unpublished observations), suggesting the intracellular targets of each compound and mechanism of cell death dynamics are distinct. Thus, *P*. *piscicida* can produce multiple secondary metabolites that induce phytoplankton mortality along a time-dependent continuum and with differential species sensitivity.

Perhaps this strategy is employed by marine bacteria in order to take the driver’s seat in controlling the flow of nutrients by creating nutrient patches by inducing cell lysis and thereby benefiting bacteria within the phycosphere^15^. Production of a suite of compounds with a range of targets and modalities would be advantageous for predatory bacteria, allowing bacteria to have greater control over their eukaryotic “farming” capabilities depending on the unique bacterial-phytoplankton interaction and surrounding environmental conditions. Additionally, the manner in which phytoplankton die has an impact on the flow and fate of nutrients^50^. We have only recently recognized viral infection and programed cell death (PCD) as important removal processes that are evoked to explain the high lysis rates of natural phytoplankton populations^50^. Our data suggest that bacterially-induced chemical death should also be a variable that one needs to consider in the phytoplankton mortality equation.

## Methods

### Phytoplankton Culturing and Enumeration

Cultures of *Emiliania huxleyi* (Plymouth Algal Culture Collection: DHB 624), *Heterosigma akashiwo* (National Center for Marine Algae and Microbiota (NCMA) CCMP 2890), *Rhodomonas* sp. (isolated from the Skidaway River Estuary, GA), *Isochrysis galbana* (CCMP 1323), *Mantoniella squamata* (CCMP 480), *Thalassiosira pseudonana* (CCMP 1335) and *Thalassiosira oceanica* (CCMP 1005) were used in experiments. Phytoplankton cultures were grown in 0.2 µm sterile-filtered, autoclaved seawater (FSW), enriched with f/2 (*T*. *pseudonana* and *T*. *oceanica*) –si (all other species) media^63^. All cultures were maintained on a 12:12 h light:dark cycle at 18 °C, salinity of approximately 30 psu, and light intensity of 85–100 µmol photons m^−2^ s^−1^. Hereafter, these conditions will be referred to as general culturing conditions. Cultures were transferred every 7–10 d to maintain exponential growth.

All phytoplankton counts were made by a flow cytometer (Guava, Millipore). Cell abundance was determined by using species-specific settings determined based on red fluorescence (692 nm) and side scatter for each species examined.

### Bacterial Culturing and Extract Production

A pure culture of *Pseudoalteromonas piscicida* (Isolate ID A757; Genbank Acc. No. KM596702) was obtained from a cryopreserved stock (10% DMSO) and 100 µL was used to inoculate multiple “starter” cultures of 8 mL of TSW media (1 g tryptone in 1 L of 75:25 seawater/MilliQ water). The starter culture was incubated at 23 °C at 100 rpm for 72 h. After 3 days, 2 mL of “starter” culture was used to inoculate seven, 1.5 L Fernbach flasks of TSY media (1 g tryptone, 1 g yeast extract in 1 L of 75:25 seawater/MilliQ water), and the newly inoculated cultures were grown at 100 rpm for 8 d at 23 °C. On day 7, 20 mL (approximately = 7.8 g) of 1:1 mixture of sterile Amberlite® XAD-7 and XAD-16 resin that had been extensively washed in organic solvent, dried, and autoclaved was added to each 1.5 L culture. Twenty-four hours later (day 8), the resin was filtered from the bacterial culture through stainless steel cloth under gentle vacuum, desalted, pooled, and allowed to dry overnight at room temperature. Secreted bacterial metabolites were eluted from the resin first in 800 mL of (1:1) methanol (MeOH): dichloromethane (DCM), followed by a second extraction in 800 mL of methanol. Both extracts were combined and the crude extract was dried under vacuum centrifugation and stored at −85 °C until testing in phytoplankton growth assays.

### Bioassay-Guided Fractionation and Isolation of Tetrabromopyrrole

As detailed in Harvey *et al*. 2016, the growth rate of *E*. *huxleyi* (strain 624) was first measured in response to live *P*. *pisicidia* cells in order to first evaluate toxicity of the bacteria to *E*. *huxleyi*. Then *E*. *huxleyi* cells were added (10^5^ cell mL^−1^ final concentration) to 24-well plates in triplicate and exposed to crude and semi-purified fractions of the bacteria extract dissolved in DMSO (0.2% v/v), in order to determine the causative mortality agent. Controls of f/2 –si media only and DMSO only were also run in triplicate with each bioassay. All plates were incubated under general culturing conditions, and enumerated on the flow cytometer. Growth rate was calculated by using the exponential growth equation, Growth rate = ln(A_f_/A_i_)/T_f_ − T_i_ where A is the abundance and T is the time over the first 72 h of the experiment. Statistical comparisons of growth rates resulting from exposure to the semi-purified fractions were compared to controls using a one-way analysis of variance (ANOVA) and Tukey’s HSD *post hoc* analysis. All statistical analyses were performed using MatLAB. Those treatments where the growth rate was significantly different from the algae only control (p < 0.05) were considered to have activity.

To create the fractions, the crude extract totaling 1260 mg was applied to a silica gel column and eluted with a step-gradient of isooctane, (4:1) isooctane/ethylacetate (EtOAc), (3:2) isooctane/EtOAc, (2:3) isooctane/EtOAc, (1:4) isooctane/EtOAc, EtOAc, (1:1) EtOAc/MeOH, and 100% MeOH, yielding eight fractions. The active fractions eluted with (4:1) isooctane/ethylacetate (EtOAc), (3:2) isooctane/EtOAc from the silica column and totaled 19.83 mg. The active fraction from the silica column was resuspended in 1:1 water/acetonitrile and further separated via an automated fraction collector into a 96-deep well plate by semipreparative HPLC using an Agilent 1200 series HPLC and a Phenomenex Kinetex 5 μm C18 100 Å (150 mm × 10 mm) column, heated to 30 °C, with a gradient of acetonitrile and water (solvents containing 0.1% formic acid) with a flow rate of 4 mL min^−1^. An active fraction comprised of a single peak at 210 nm eluted with (3:1) acetonitrile/water. A total of 3.2 mg of active compound, determined to be tetrabromopyrrole (TBP), was recovered from 10.5 L of bacterial culture after 8 days of growth, yielding a final concentration of 0.79 μM (0.25% of crude). An authentic TBP standard was obtained from Bradley Moore (UCSD) and used in subsequent phytoplankton inhibition assays.

Accurate mass spectra for TBP was acquired on an Agilent Technologies 6230 ToF with a Dual Agilent Jet Stream Electrospray Ionization source, equipped with an Agilent 1260 Infinity series HPLC containing a Phenomenex Kinetex 2.6 μm C18 100 Å (150 mm × 2.1 mm) column as the stationary phase with a flow rate of 0.2 mL min^−1^. The ion source was operated at 350 °C and 3500 V with a nitrogen gas flow of 8 L min^−1^, nebulizer pressure of 40 psi and fragmentor voltage of 135 V. The chromatography method was as follows: starting conditions were 9:11 water/acetonitrile; then ramped to 3:7 water/acetonitrile over 10 min; held at 3:7 water/acetonitrile for 3 min; ramped to 1:20 water/acetonitrile for 1 min; held at 1:20 water/acetonitrile for 6 min; then returned to 9:11 water/acetonitrile and held for 3 min. All solvents were acidified with 0.1% formic acid. The instrument was equipped with an Agilent Mass Hunter Workstation version B0.4.00 software.

### Phytoplankton Inhibition Assays

The dose-response relationship of eight phytoplankton species (listed above) to TBP was measured by exposing cells from exponentially growing cultures to a gradient of TBP concentrations in triplicate in a 24-well plate. Well plates were kept under general culturing conditions, and cell abundances were monitor daily via flow cytometry. Similar inhibition assays were conducted for 8 other pure compounds in order to examine how chemical structure and bromination influenced phytoplankton growth dynamics.

Percent of algal survival was plotted against the concentration of each pure compound to determine the concentration of compound resulting in 50% growth inhibition (IC_50_). Pure compound IC_50_ values were calculated and 95% confidence intervals were estimated using Prism 6.0 software (GraphPad) by fitting the log transformation of the response variable (I; inhibitor concentration) by non-linear regression to the equation, Y = Bottom + (Top – Bottom)/ (1 + 10^((LogIC^_50_^−I)*Hill Slope)^), where the slope factor (Hill Slope) is equal to -1.0 and the “Top” and “Bottom” numerals equal the plateaus of curve in units of percent survival. Significance between two IC50 values were determined using an extra sum of squares F test^64^.

Recovery assays were conducted with *E*. *huxleyi* to investigate the recovery of algal cells to previous TBP exposure. Cells were exposed to the compound as described above in an inhibition assay. After 96 h of exposure, *E*. *huxleyi* cells from all concentrations were washed 3x by gentle centrifugation (4000 rpm for 5 min), and re-suspended in f/2 –si media. Abundance of these TBP-free cultures were monitored via flow cytometry for 72 h, and the growth rate was calculated as described above. Cells that were exposed to no compound and to DMSO-only served as controls; all recovery assays were replicated in triplicate.

### Reactive oxygen species (ROS) production & intracellular calcium signaling

Flow cytometry was used to examine the role of TBP in influencing calcium signaling and reactive oxygen production (ROS). To examine calcium signaling, 200 µL of an exponentially growing *E*. *huxleyi* culture was incubated with 2.5 µM Fluo-4 AM dye (494/506 nm) and then aliquoted into a 96 well plate. The experimental agonist, ionomycin, was added at a final concentration of 16 µM in the positive control trial and the final concentrations of TBP in the experimental trials were between 1–100 nM. Stained and unstained *E*. *huxleyi* cells with no TBP added served as controls, and all treatments were run in triplicate. Once the dye was added, cells were incubated in the dark at 18 °C for 90 min, and then run on the flow cytometer every 5 min for 30 min total.

For the measurement of intracellular ROS production, 200 µL of an *E*. *huxleyi* culture was treated with 5 µM of the CM-H_2_DCFDA stain and 1–100 nM of TBP. Cells were incubated in the dark at 18 °C for 90 min, and then run on the flow cytometer every 10 min for 1 h, and then a final measurement was made after 2 h of exposure. Similar to the calcium tests, stained and unstained *E*. *huxleyi* cells with no added TBP served as controls, all treatments were run in triplicate.

Significant differences for both tests were determined by comparing the percentage of the population that was stained in each treatment to the DMSO control using a repeated measures ANOVA, followed by a Dunnett’s multiple comparisons test^65^.

### Exploring the biogeography of TBP biosynthesis genes

A conserved four-gene locus encoding the enzymes involved in TBP biosynthesis was previously identified from *P*. *piscicida* (A757) and used to search the Ocean Gene Atlas web-based platform to explore the biogeography of genes with homology to TBP biosynthesis enzymes. Protein sequences from *P*. *piscicida* (A757) (Genbank Accession numbers: AME30284.1 to AME30287.1) were searched against the Ocean Microbial Reference Gene Catalog (OM-RGC, version 1) using BLASTp search tools with an initial customized e-value threshold of 1.0e^−10^. Further stringency in e-value thresholds was determined by manually looking at the data and underlying sequences to confirm similarity to reference genes. The quantitative distribution of environmental sequences with similarities to *bmp1* to *bmp4* genes were analyzed by geographic distribution, co-variation with environmental features, and taxonomic distribution. Quantitative distributions of homologous sequences were mapped using filled circles with sizes proportional to their combined abundance at three sampling depths (subsurface, deep chlorophyll maximum, and mesopelagic) and the circle colors indicated the size fractionation applied to the sample.

## Electronic supplementary material

Supplementary Information

## Data Availability

Biosynthetic gene sequences for TBP can be found in Genbank with accession numbers: AME30284.1 to AME30287.1.
