# Supplementary material for: The chemical cue tetrabromopyrrole induces rapid cellular stress and mortality in phytoplankton
Source: Sci Rep. 2018 Oct 19;8:15498. doi: 10.1038/s41598-018-33945-3 (PMC6195506; doi:10.1038/s41598-018-33945-3)
Supplement: Supplementary file 1 — Supplementary Information [file 41598_2018_33945_MOESM1_ESM.pdf]

Supplementary Information for

The chemical cue tetrabromopyrrole induces rapid cellular stress and mortality in phytoplankton

Kristen E. Whalen[1*], Christopher Kirby[2], Russell M. Nicholson[1], Mia O'Reilly[1], Bradley S. Moore[3], Elizabeth L. Harvey[2*]

[1]Department of Biology, Haverford College, Haverford, PA, USA
[2]Skidaway Institute of Oceanography, University of Georgia, Savannah, GA, USA
[3]Scripps Institution of Oceanography, University of California at San Diego, La Jolla, CA, USA

Corresponding Authors:
Kristen Whalen, 370 Lancaster Ave, Haverford, PA 19041
kwhalen1@haverford.edu

Elizabeth Harvey, 10 Ocean Science Circle, Savannah, GA 31411,
elizabeth.harvey@skio.uga.edu

This PDF file includes:

Table S1
Figs. S1 to S3
References for SI reference citations

Table S1 – Inhibitory concentrations (IC_{50}) from seven species of phytoplankton after 24 and 48 hrs of exposure to TBP. Values in parentheses are one standard error of the fit of the curve to the data points. P-values are testing for significant differences in calculated IC_{50} values between time points of expsoure. After 24 h of exposure, *T. pseudonana* exhibited a response to TBP at >5225 nM only, therefore an IC_{50} value could not be calculated, nor could a comparison to 48 h IC_{50} values be conducted.

	**24 h**				**48 h**				
**Species**	**IC_{50} (ng mL^{-1})**	**IC_{50} (nM)**	**R^2**	**95% CI (nM)**	**IC_{50} (ng mL^{-1})**	**IC_{50} (nM)**	**R^2**	**95% CI (nM)**	**p value**
Mantoniella sp.	27 (3)	72 (8)	0.98	51 - 107	23 (5)	62 (13)	0.93	32 - 117	0.65
Emiliania huxleyi	21 (2)	57 (5)	0.94	38 - 83	36 (2)	94 (5)	0.89	60 - 151	0.42
Isochrysis galbana	108 (6)	291 (15)	0.85	94 - 884	55 (6)	144 (15)	0.88	55 - 374	0.43
Thalassiosira oceanica	194 (2)	507 (6)	0.91	228 - 1124	72 (4)	188 (12)	0.83	57 - 616	0.32
Heterosigma akashiwo	84 (2)	220 (6)	0.92	101 - 478	86 (3)	224 (8)	0.89	91 - 548	0.96
Rhodomonas sp.	390 (6)	1051 (15)	0.94	533 -2064	93 (7)	243 (18)	0.91	105 - 566	0.01
Thalassiosira pseudonana	-	>5225	-	-	109 (2)	286 (5)	0.93	141 - 578	-

Supplementary Figures

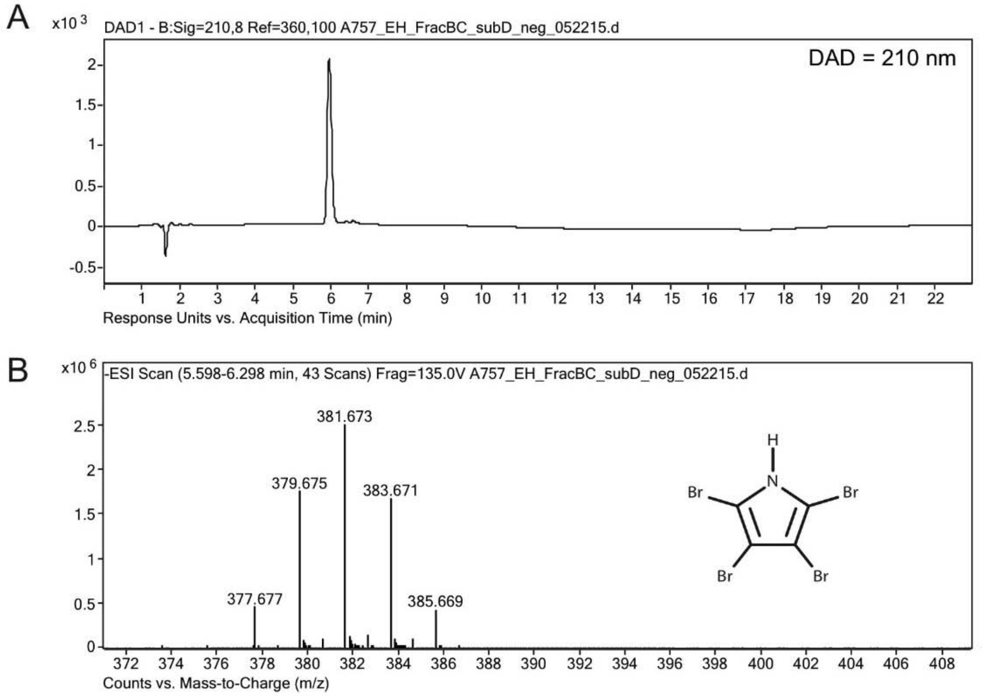

Fig. S1. Composite figure showing TBP structural elucidation from isolate A757, (A) HPLC separation of the active constituent from *P. piscicida* (A757) visualized at 210 nm. (B) Mass spectrum [(-)-HRESI] of the active constituent from *P. piscicida* (A757; structure of tetrabromopyrrole in inset).

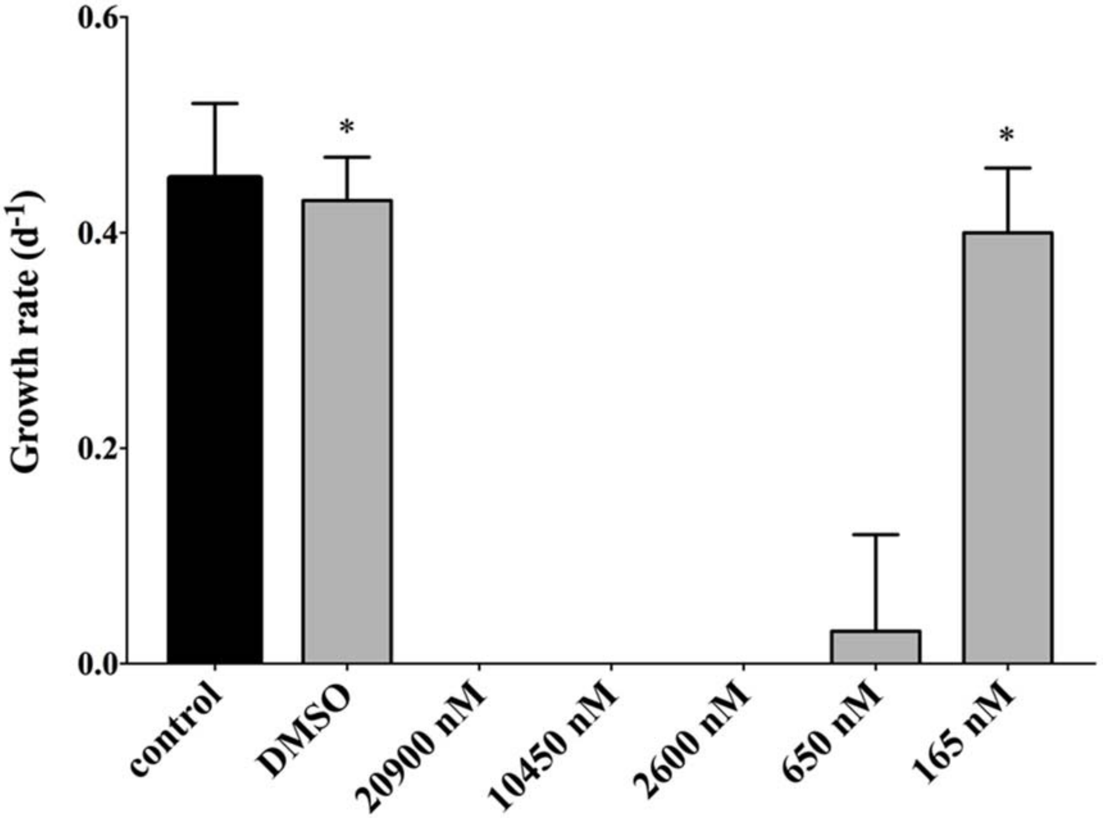

Fig. S2. The 72-hr growth rate of *E. huxleyi* in TBP-free media, after being exposed to TBP concentrations, between 165 and 20,900 nM, for 96 hrs. Controls included a DMSO addition and cells-only (black bar) treatments. Only *E. huxleyi* exposed to 165 nM of TBP recovered with similar growth rates to the cells-only and DMSO controls (asterix indicate no significant different to the cells-only control). Each symbol represents the mean of three independent replicates +/- the standard deviation.

Fig. S3. Genes with homology to pseudoalteromonad TBP biosynthesis genes are predominantly coastal in their distribution. The Ocean Gene Atlas (1) was mined using protein sequences from *P. piscicida* (A757) *bmp1* to *bmp4* genes, shown to be involved previously in TBP synthesis (2). The circle diameter indicates the percent of genes within an environmental sample matching the query which ranges from 0.000142% to 0.00000000148% with the e-value cutoffs of 1.0e^{-15}, 1.0e^{-43}, 1.0e^{-55}, 1.0e^{-50}, for *bmp1 to bmp 4,* respectively. Circle color indicates the size fractionation applied to the sample. Gene abundances with homology to each query are shown for subsurface (SRF), deep chlorophyll maximum (DCM), and mesopelagic (MES) depths.

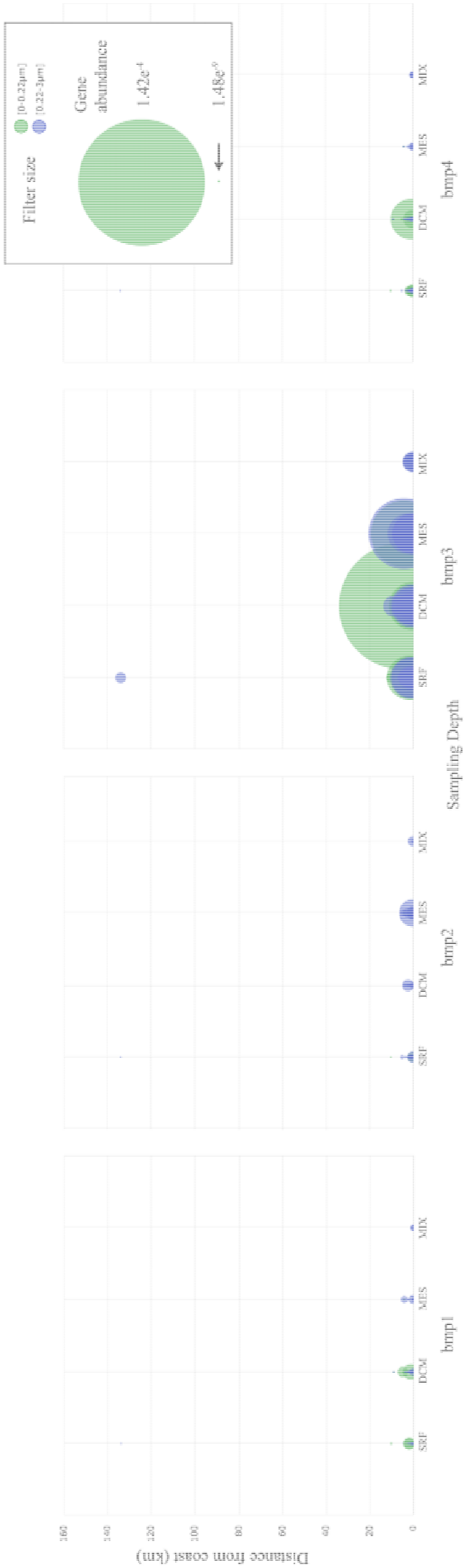

References

1. Villar E, *et al.* (2018) The Ocean Gene Atlas: exploring the biogeography of plankton genes online. *Nucleic Acids Res*. 46(W1):W289-W295. doi: 10.1093/nar/gky376.

2. El Gamal A, *et al.* (2016) Biosynthesis of coral settlement cue tetrabromopyrrole in marine bacteria by a uniquely adapted brominase-thioesterase enzyme pair. *Proceedings of the National Academy of Sciences of the United States of America* 113(14):3797-3802.
